# A Unified Framework for Reservoir Computing and Extreme Learning Machines based on a Single Time-delayed Neuron

**DOI:** 10.1038/srep14945

**Published:** 2015-10-08

**Authors:** S. Ortín, M. C. Soriano, L. Pesquera, D. Brunner, D. San-Martín, I. Fischer, C. R. Mirasso, J. M. Gutiérrez

**Affiliations:** 1 Instituto de Física de Cantabria, CSIC-Universidad de Cantabria, E-39005 Santander, Spain; 2 Instituto de Física Interdisciplinar y Sistemas Complejos, IFISC (CSIC-UIB), Campus Universitat Illes Balears, E-07122 Palma de Mallorca, Spain; 3 Predictia Intelligent Data Solutions S.L., E-39011 Santander, Spain

## Abstract

In this paper we present a unified framework for extreme learning machines and reservoir computing (echo state networks), which can be physically implemented using a single nonlinear neuron subject to delayed feedback. The reservoir is built within the delay-line, employing a number of “virtual” neurons. These virtual neurons receive random projections from the input layer containing the information to be processed. One key advantage of this approach is that it can be implemented efficiently in hardware. We show that the reservoir computing implementation, in this case optoelectronic, is also capable to realize extreme learning machines, demonstrating the unified framework for both schemes in software as well as in hardware.

A number of machine learning techniques using random nonlinear projections of inputs into a high-dimensional space have been proposed during the last decade^1^,^2^. In case of using recurrent networks for the projection, they are sometimes referred to as reservoirs. Outputs are typically obtained as linear combinations of the reservoir node states with weights trained from data in a supervised manner, thus strongly simplifying the training process. Two of the most popular random-projection techniques are Extreme Learning Machines (ELMs)^3^ and Reservoir Computing (RC) and in particular Echo State Networks (ESNs)^4^. Both concepts have been developed independently, including different terminology and notations^5^. The ELMs were introduced as a simplification of (one-layer) feedforward neural networks, suitable for prediction and classification problems. The ESNs were inspired in recurrent neural networks, suitable for time dependent data.

In this paper we propose a unified framework for random-projection machines, based on ESNs and ELMs. Although the similarities between both concepts are now being recognized^1^,^6^, this is the first time that ELMs and ESNs are implementated on identical hardware. This fact illustrates the strong conceptual analogies between the two approaches.

We present an implementation of both ELM and ESN in the same hardware where the switching between the two concepts (ESNs and ELMs) is easily obtained by activating or deactivating one physical connection. We build on a recently proposed architecture for RC consisting of a single nonlinear neuron subject to a recurrent self-feedback loop^7^,^8^,^9^,^10^,^11^. An advantage of the proposed architecture is that it can be easily implemented in hardware, potentially allowing for high-speed information processing. In this particular case, we choose an optoelectronic system similar to those described in^8^,^9^. We would like to highlight that our approach is not bound to this particular architecture. It can be easily extended to any hardware implementations of a single neuron with a delay feedback line, including electronic and all-optical implementations^7^,^8^,^9^,^10^,^11^. In these cases, the ESN system can be turned into an ELM by simply opening the recurrent feedback loop.

The paper is organized as follows. First, we present the unified framework for ESNs and ELMs. Second, we describe the implementations of the unified framework using a single neuron subject to a self delay-feedback loop in software and hardware, respectively. Then, we present the numerical and experimental results for several benchmark tasks. Finally, we provide some concluding remarks.

## Unified framework

Both ELMs and ESNs belong to the category of supervised learning machines, aimed at learning from data the conditional expected value of a *m*-dimensional output **y**(*n*) as a function of a *d*-dimensional input **x**(*n*), based on a number of examples *n* = 1, …, *N*. The particularity of these approaches is the use of an intermediate reservoir space, where inputs are randomly projected in a nonlinear form, yielding a new, transformed *D*-dimensional **r**(*n*) space (typically *D* ≫ *d*). In the following we shall refer to the elements of the reservoir space as neurons, since these approaches were initially developed in the framework of neural networks and neuron-like functions were used to perform the nonlinear transformations. These models can be written in the following general form^1^,^3^,^4^:


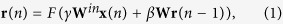



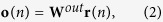


where **r**(*n*) and **o**(*n*) are the state of the reservoir neurons and the predicted output values, respectively. **W**^*in*^ is the *D* × *d* (random) input weight matrix that projects the input onto the reservoir, *F* is the reservoir nonlinear activation function (typically a sigmoid function), **W** is the *D* × *D* matrix that denotes the reservoir neuron connectivity and *γ* and *β* are input and connecting scaling factors, respectively. The values of **W**^*in*^ and **W** are usually drawn from a uniform distribution over [1, −1]. In ELMs there is no inter-neuron connectivity^3^ and matrix **W** therefore is zero. In ESNs, the connectivity matrix is typically sparsely filled, providing memory for problems with temporal structure in the input data^4^. Finally, **W**^*out*^ are the output weights of the linear combiner that are computed by minimizing the squared error between the actual **y**(*n*) and predicted **o**(*n*) output values. Different regularization techniques can be used to obtain **W**^*out* ^12,13. The ridge regression technique is one of the most popular in ESNs^12^, whereas the Moore-Penrose pseudo-inverse technique is applied in classical ELMs^13^. In this paper, we use the Moore-Penrose pseudo-inverse following the classical ELM approach for comparability. Our numerical and experimental implementations include several sources of noise that already lead to stable models.

**Figure 1:**
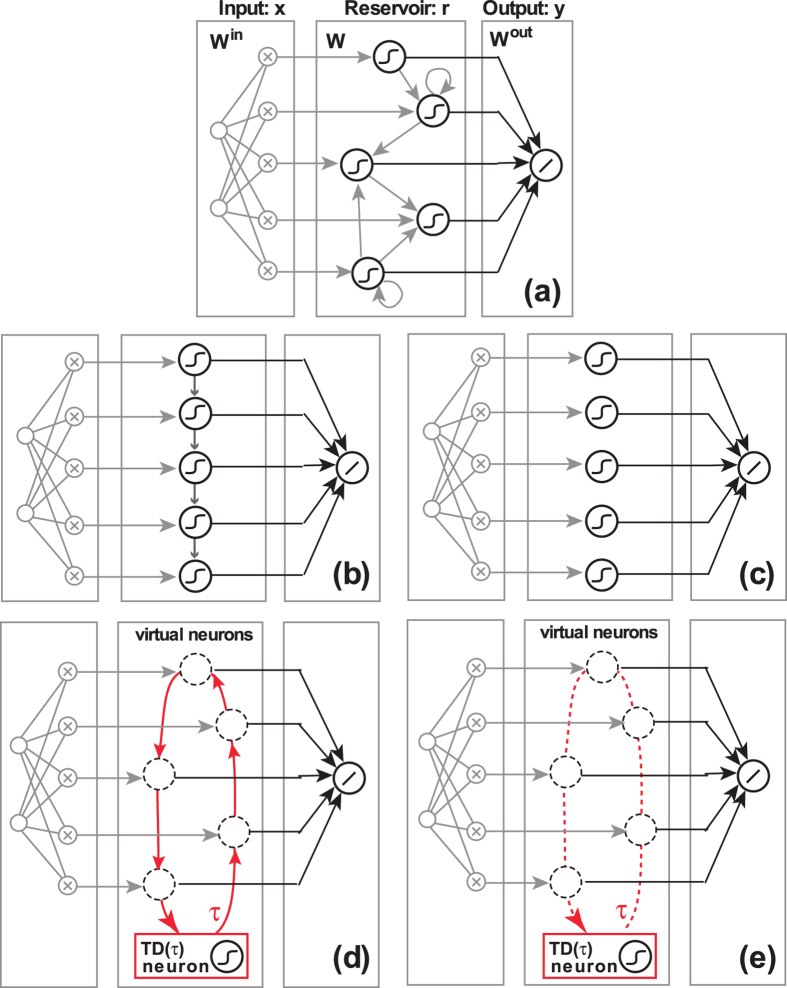
Schematic illustration of different types of random-projection machines: (a) original ESNs, with sparse inter-neuron connectivity, (b) ESNs with simple chain connectivity, and (c) ELMs, with no connectivity. Panels (**d**,**e**) are equivalent to (**b**,**c**), respectively, but considering a single neuron with delay as nonlinear processor. The virtual neurons (virtual nodes), circles with dashed lines in (**d**,**e**), are addressed via time-multiplexing and form a reservoir. The dashed arrow in (**e**) indicates that the virtual neurons (virtual nodes) are time-multiplexed versions of the single neuron with delay but are not connected among them through the feedback loop. Weights trained during the learning process are indicated by black arrows, whereas predefined (or random) weights are depicted with gray or red arrows.

This general form is schematically represented in Fig. 1(a). Although it is not explicitly stated in the figure, the *d*-dimensional input **x** is augmented with an additional constant neuron accounting for the bias term. Learning from data is efficiently achieved through the random projection “trick”, since the only weights to be trained in this approach are those corresponding to the reservoir-output connections, **W**^*out*^ (shown in black color in the figure).

In general, Eq. 1 works as an ESN when the parameter *β* is adjusted to ensure the system has fading memory (the echo state property)^14^. In this case, the connection matrix **W** is usually a sparse random matrix. It has recently been shown that simpler and deterministic connection topologies perform as well as standard random connection matrix^15^,^16^. Particularly interesting are ESNs with a simple chain (or ring) topology (i.e. connection matrix with only non-zero elements on the lower sub-diagonal). This particular ESN configuration is schematically illustrated in Fig. 1(b), and for a particular reservoir neuron *i* corresponds to the following simplification of Eq. 1^15^:


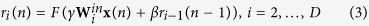


with 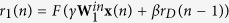 or 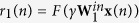 for ring and chain topologies, respectively. Note that 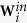 refers to the *i*th-row of the input matrix **W**^*in*^.

In contrast, ELMs correspond to the simplification of (1) or (3) with no inter-neuron connectivity (*β* = 0). This is schematically represented in Fig. 1(c).

### Implementation with a single time-delay neuron

Recently, it has been proven that (3) can be efficiently implemented considering a single nonlinear neuron (or more general a nonlinear dynamical element) with a feedback loop^7^, which is governed by:


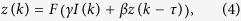


where *τ* is the delay time and *β* and *γ* are the feedback and input scaling parameters, respectively. *I*(*k*) is the generic input that drives the system. The *d*-dimensional input is sequentially processed by driving the feedback-coupled neuron using time-multiplexing. Thus, the nonlinear projected reservoir space is composed of the *D* consecutive outputs of the system, **r**(*n*) = (*z*(*nD* + 1), …, *z*(*nD* + *D*)). In this way, the spatial multiplexing of the input in the systems with *D* neurons is replaced here by time-multiplexing. These *D* points are called virtual neurons since they play the same role than the neurons in the standard implementation. In the case of *τ* = *D* + 1^8^, the virtual neurons, *r*_*i*_(*n*) = *z*(*nD* + *i*), can be described by:


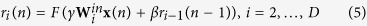


and 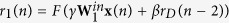. For all practical purposes, this approach is equivalent to the ESN defined by equation (3) with ring topology.

Interestingly, simply by removing the feedback line and thereby setting *β* = 0, the virtual neuron-states are only the response of the system to the injected information, 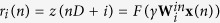. Such a system is completely identical to the ELM case (see equation (3) with *β* = 0). One can therefore easily implement an ELM by the same setup employed for the implementation of an ESN.

Schematic diagrams of the single feedback neuron realizing RC (ESN) and ELM are plotted in Fig. 1(d,e), being equivalent to the standard models shown in panels (b) and (c), respectively. In fact, the only difference between panels (d) and (e) in Fig. 1 is that the network connections were removed. Such modifications to the connectivity structure are easily performed in software. However, they are typically more demanding in a physical hardware implementation with many neurons. The hardware implementation of the single delay neuron with a feedback line provides a scheme where the switching between physical implementations of two concepts (ESNs and ELMs) is easily obtained by simply activating or deactivating a single hardware-connection only.

### Experimental optoelectronic setup

A single neuron with a feedback line can be easily implemented in hardware. Traditionally, ESN and ELM are realized in software by using networks of neurons. In the delay-based reservoir, there is a single neuron and a collection of virtual neurons in the delay line. From now on, these virtual neurons will be called virtual nodes.

The general equation that governs these delay-systems is:


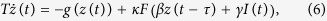


where *T* is the response time of the system, *τ* is the delay time, *I*(*t*) is the external input, *γ* is the input scaling, *κ* is the nonlinearity gain, *β* is the feedback-strength, *F* and *g* are nonlinear and linear functions, respectively.

To build this architecture it is necessary to go from the discrete time of the original input **x**(*n*) to a continuous time implementation. The external input *I*(*t*) is the continuous version of the discrete random projection of data **W**^*in*^**x**(*n*). To construct this continuous data *I*(*t*), **W**^*in*^**x**(*n*) is multiplexed in time^7^,^8^. For each value of **x**(*n*), the data injected into the nonlinearity undergoes a sample-and-hold procedure, during which the injected value is kept constant for duration *θ*. In this way, 
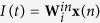
 holds for the time interval *θ*(*nD* + *i* − 1) < *t* < *θ*(*nD* + *i*).

As a response to input **x**(*n*), the state of the virtual nodes forming the reservoir are defined by *r*_*i*_(*n*) = *z*(*nDθ* + *iθ*), where *i* = 1, …, *D*. Thus, it takes a time *θD* to obtain the reservoir state for the original input **x**(*n*). Choosing a *θ* > *T*, where *T* is characteristic-timescale of the hardware neuron, the state of the virtual nodes are practically independent of the states of the neighboring virtual nodes, and the experimental implementation works as an ELM if *β* = 0 or as an ESN with ring topology (see Eq. 5) if *τ* = *θ*(*D* + 1).

**Figure 2:**
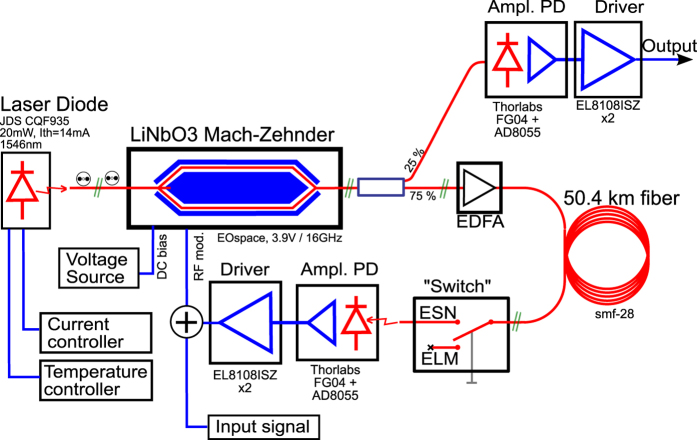
Experimental implementation of either an ELM or an ESN in photonic hardware. The nonlinear projection is provided by a Lithium-Niobate Mach-Zehnder modulator, modulating the intensity of a standard semiconductor laser-diode. Simply by using a fiber-switch, one can select the information injected into the modulator to be *γI*(*t*) for the case of an ELM-implementation, or to be *βz*(*t* − *τ*) + *γI*(*t*) when implementing an ESN.

Our optoelectronic hardware-implementation is schematically illustrated in Fig. 2. An integrated telecommunication Mach-Zehnder modulator (MZM, LiNbO_3_) implements a 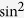 nonlinear transformation, which modulates the emission of a standard telecommunication laser-diode (20 mW), emitting at 1550 nm. A long optical fiber (50.4 km) combined with an electronic feedback circuit implements the delayed feedback loop, with the feedback-delay being *τ* = 247.2 *μ*s. The electronic circuit acts as a low pass filter, with a characteristic response time of *T* = 240 *ns*. The experimental set-up has a switch that controls if the recurrent feedback loop is open or closed. If the switch closes the feedback loop, the circuit allows to combine the input information *γI*(*t*) with the delay signal *βz*(*t* − *τ*) and the system works as an ESN. The signal is amplified to allow for sufficiently nonlinear operation. If the switch does not close the feedback loop then the nonlinear operation is only performed over the input information *γI*(*t*) and the implementation works as an ELM. Our experimental system provides direct access to key parameters, e.g. the nonlinearity gain *κ* and the offset phase of the MZM *ϕ*, enabling easy tunability of nonlinearity and dynamical behaviors. The product *κβ* is equivalent to the spectral radius in ESNs^12^,^14^. The parameter *κ* is controlled via the laser diode power, while *ϕ* is controlled by the DC-bias input of the MZM. Further details about the experimental setup can be found in^9^ where a similar implementation was used as a reservoir computer. Much higher processing speed, reaching 10 GSamples/s, have already been achieved by a comparable system^17^.

The random mapping provided by our experimental setup is given by


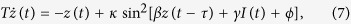


with *ϕ* being the phase of the system. In absence of input (*γ* = 0) and *κβ* < 1, the system is stable and the steady state of *z* depends on the offset phase *ϕ*.

The experimental reservoir state *z*(*t*) is obtained from a fraction of the optical signal at the output of the Mach-Zehnder, which is detected with a photodiode. The electronic signal of the photodiode then passes an analog-to-digital converter (ADC) that has a limited bit-resolution. Several noise sources affect the signal recorded from this optoelectronic system. However, the major sources of noise-induced performance degradation in the optoelectronic setup are the quantization and classifier-detection noise^18^. These two noise sources originate in the detection process and can be modelled by including an additive Gaussian noise term in the right-hand side of Eq. 7 and by quantizing the resulting value of the states *z*(*t*).

## Results

In this section we obtain the performance of ESNs and ELMs for several tasks using the unified approach based on the single neuron with a feedback line shown in Eq. 5. Our main aim is to gain insight into the behaviour of the system and to analyze the similarities and differences between ESN and ELM approaches. To this end, we consider several benchmark tasks: memory capacity, one-step ahead time-series prediction of a Mackey-Glass system, channel equalization and cancer classification via DNA-microarray data. While the memory capacity is an academic application that characterizes the fading memory of the system and allows us to gain insights into the capability of the system to process history-dependent information, the channel equalization task represents a more technologically relevant application. Complementarily, the Mackey-Glass time-series prediction and cancer classification pose different challenges for machine learning; the former is based on a low dimensional input with a large training dataset (*d* ≪ *N*), whereas the latter is based on a high-dimensional input with a small training dataset (*d* ≫ *N*).

We test the performance of the optoelectronic setup described in the experimental section when it is used as ELM or ESN and compare the experimental results with the numerical counterparts. The simulation results are obtained by numerically solving Eq. 5 with 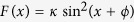 and *β* = 1 for ESN or *β* = 0 for ELM. Since noise is an intrinsic component of the hardware system, we also provide a sensitivity analysis to test the influence of the noise-induced performance degradation in the optoelectronic setup.

### Memory capacity

First, we evaluate the memory capacity of the system. The memory capacity is a task designed to estimate the fading memory of reservoir computers^14^, i.e. the temporal information about the input that the system can remember after a certain time. To compute the memory capacity, the input is an identically distributed random sequence, *y*(*n*), uniformly distributed between [−1, 1]. The desired output values are delayed versions of the input signal *y*(*n* − *i*). The memory function, *m*(*i*), is given by the normalized linear correlation between the desired outputs and their associated predicted outputs from the system *o*_*i*_(*n*)^14^:


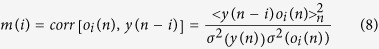


where < >_*n*_ is the mean over all values *n* and *σ* denotes the standard deviation. The memory capacity *MC* is then defined as the sum of the memory function *m*(*i*), with *i* going to infinity^14^:


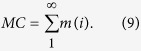


We compute the memory function for the ESN and the ELM. Both, for the numerical and experimental results, the system parameters are *κ* = 0.9, *ϕ* = 0.4*π*, *D* = 246 and 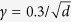, where *d* is the input dimensionality. We use 4000 samples for training, 3000 samples for validation and 3000 samples for testing. The system output to the first 100 inputs is discarded to avoid transient periods. The numerical results are the average over 10 runs with different training/validation/testing sets and random input weights ***W***^*in*^.

**Figure 3:**
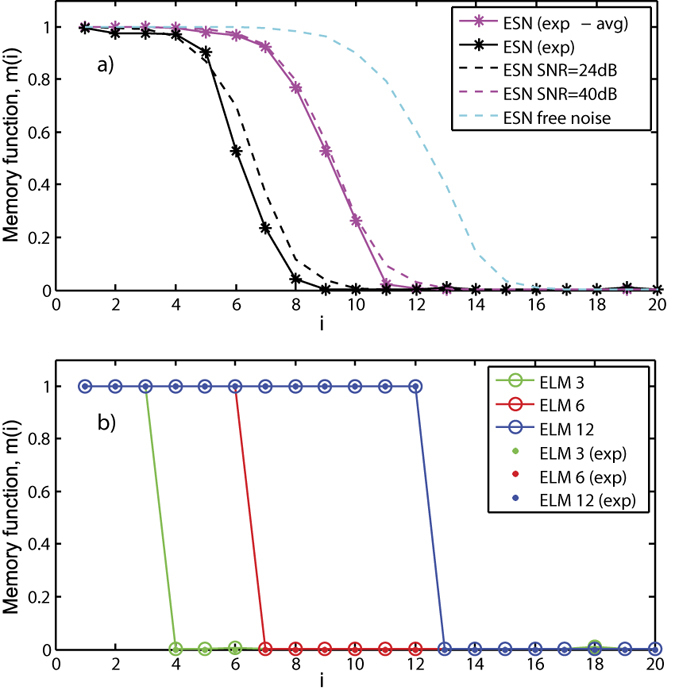
Memory function for the numerical (dashed lines) and experimental (solid lines) realizations of (a) ESN with *d* = 1 and (b) ELM with *d* = 3, 6 and 12, respectively.

In order to compare the experimental and numerical results, we measure the signal to noise ratio of the experimental realizations and use the corresponding system and quantization noises in the numerical simulations. We find that the signal to noise ratio (SNR) of a single measurement is ~24 dB, although the experimental SNR can be increased to 40 dB by averaging the detection over ten repetitions of the measurement. Figure 3(a) shows the memory function, *m*(*i*) for the ESN implementation. We find excellent agreement between numerics and experiments. Experimental memory functions with SNR ≈40 dB and SNR ≈24 dB yield a memory capacity of *MC* = 8.5 and 6, respectively. Since noise degrades the memory capacity of the system, we also show the numerical results without noise, which yield a MC around 12. The memory capacity of the noise-free system is approximately twice the MC of the system with 24 dB noise.

ESNs have an intrinsic memory of the past inputs due to the reservoir-connectivity. In the ESN case, a scalar input *x*(*n*) = *y*(*n*) is used to calculate the memory capacity. In contrast, ELMs are memory-free learning machines and the required input information has to be provided explicitly in the form of a *d*-dimensional input vector (*x*_1_(*n*), …, *x*_*d*_(*n*)) = (*y*(*n* − 1), …, *y*(*n* − *d*)) created by temporal multiplexing. Figure 3(b) shows the memory function for the experimental and numerical realizations of the ELM with *d* = 3, 6 and 12. Experiments and simulations give the same results. The memory function of the ELM implementation is a step-wise function with *m*(*i*) = 1 if *i* ≤ *d* and zero otherwise. Thus, the *MC* = *d* and the ELM does not have more information about past inputs except to the one provided by the input. Therefore, ELMs have the disadvantage of requiring extra information to specify the appropriate number of inputs required by the task. In contrast, unlike the ESNs, the influence of system noise in the ELM memory capacity is negligible because the memory is explicitly provided in the input data. Due to this reason ELM is also less noise prone than ESN for memory dependent tasks such as the Mackey-Glass time-series prediction task.

The memory capacity of ELMs is directly provided by the input data and therefore it is totally independent of the system parameters. On the contrary, the memory capacity of the ESNs is intrinsic to the system and changes with the nonlinearity response of the ESN. Almost linear reservoir computers give the highest MC but in return they can not carry on nonlinear computations. Thus, there is a clear compromise between the MC and the nonlinear computation capacity in RC^19^. In our particular implementation of ESN, the nonlinearity of the system is determined by the system phase, *ϕ*, and the input scaling factor. The maximum MC is obtained at *ϕ* = 0.1*π* and *ϕ* = 0.6*π*, the system phases that lead to steady states at the most linear part of the Ikeda nonlinearity. At these phases and with *γ* = 0.3, the numerically obtained MC are 13 and 20 with a SNR ≈24 dB and the noise-free system, respectively. In the noise-free case, lower input scaling factors yield higher MC but also imply a system response with a smaller amplitude. In presence of noise, there is a trade off between the MC degradation due to the reduction in the SNR and the MC increases due to the use of a lower input scaling factor.

### Mackey-Glass time series prediction

We now consider a popular example of time series prediction, obtained from the chaotic Mackey-Glass system, which is defined by the following delay differential equation:


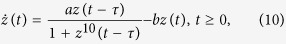


with the typical values *a* = 0.2, *b* = 0.1 and *τ* = 17. In particular we consider a discrete time series *y*(*n*) = *z*(*nT*), *n* = 1, …, 10000, obtained from the continuous system with sampling time *t*_*s*_ = 3. In this work, the time series is normalized with mean zero and standard deviation one and divided by the squared root of the input dimensionality before being used. The time series is divided into three sets where the first 4000 points are used for training, the next 3000 for validation and the last 3000 points for testing purposes.

Our aim is to perform a one-step ahead prediction of *y*(*n*) using previous values of the time series. In order to include information from the delayed component at *τ* = 17, the minimum memory required for this problem is 6 in our particular case (time-horizon *t*_*s*_ = 3 and *y*(*n*) = *z*(*nt*_*s*_)). The ESN implementation has an intrinsic memory capacity that already provides the required memory for this task as we have shown in the previous section. In this case only a scalar input *x*(*n*) = *y*(*n* − 1) is used to predict *y*(*n*). In contrast, ELMs as memory-free learning machines need a *d*-dimensional (*d* ≥ 6 for this problem) input vector (*x*_1_(*n*), …, *x*_*d*_(*n*)) = (*y*(*n* − 1), …, *y*(*n* − *d*)) created by temporal multiplexing to capture the required memory to predict the output *y*(*n*).

**Figure 4:**
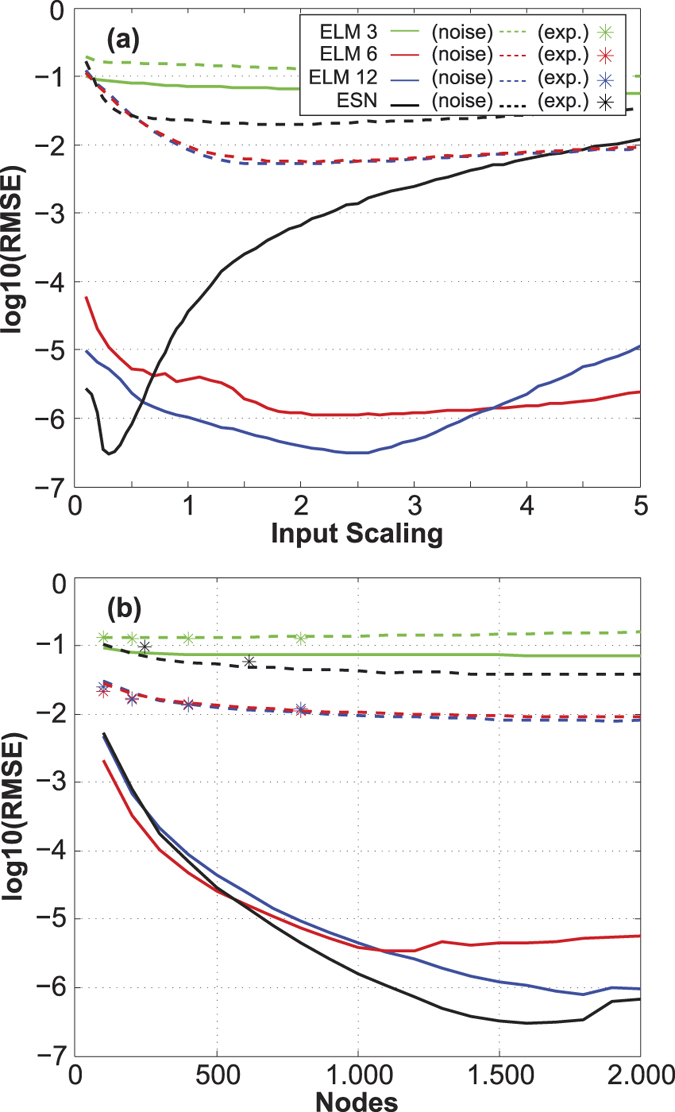
Normalized error (in logarithmic scale) of the Mackey-Glass time-series prediction for: (top) ELM and ESN machines with *D* = 1500 neurons and different input scaling values, from 0.1 to 5; (bottom) ELM and ESN models with input scalings 1 and 0.3, respectively. Results for noisy output are indicated by dashed lines.

In order to check the sensitivity of the ELM results on the number of inputs, we consider three different configurations: *d* = 3, 6 and 12, with insufficient, sufficient and excessive input information, respectively. Since the model (5) depends on two tunable parameters *γ* and *β*, which control the scaling of the input and feedback components, respectively, we start by analyzing the influence of these parameters on the results. Note that in the standard implementation of ELMs, *β* = 0 and *γ* = 1. To this aim, we fixed the values of *κ* = 0.9, *ϕ* = 0.07*π*, *D* = 1500 *β* = 0 (ELM) or *β* = 1 (ESNs) and computed the validation errors for the models resulting from different input scaling values, with *γ* ranging from 0.1 to 5. Figure 4(a) shows the normalized validation errors in logarithmic scale for three ELM configurations (with 3, 6 and 12 delayed inputs) and a ESN (only one input) as a function of the input scaling. Results obtained by adding system noise and quantization noise (resolution of 7 bits) to the reservoir values are indicated by dashed lines. This figure shows that the results for ESNs are much more sensitive with optimum performance for smaller input scaling values than for the ELMs. This can be explained by the amount of memory required by the ESN to solve the Mackey-Glass prediction task, which is a function of the input scaling, with larger memories corresponding to small input scaling^9^. However, the optimal parameter sets for the Mackey Glass and the MC are not the same because the Mackey Glass prediction task also requires nonlinear computation capacity. It is worth noting that since noise degrades the MC, in the presence of a large amount of noise the MC for the optimal phase can be close to the amount of memory required by the ESN to solve the Mackey-Glass task. In this situation the optimal phases for the Mackey Glass and the memory capacity tasks will be close to each other.

In the following we considered the values 0.3 (optimum) and 1 for the input scalings of the ESNs and ELMs, respectively. For these parameter values the MC is similar to the one shown in Fig. 3. Note that the value *γ* = 1 (used in the standard implementation of ELMs) gives close to optimum performance values, and could therefore be considered a reasonable choice for this parameter for general ELM applications. Figure 4(b) shows the test errors as a function of the number of neurons (for the corresponding optimum input scaling).

In contrast to reference^4^, the Mackey-Glass prediction task is done for a one-step ahead, which corresponds to *t* = 3 in normalized units of Eq. 10. ELMs typically use a small number of neurons to solve the Mackey Glass prediction task^20^. In Figure 4 (bottom) we show, however, that much better results are obtained in both ELMs and ESNs when a large number of nodes is used. From Figure 4 it can also easily be seen that a similar behavior between ELM and ESN is obtained when the required delayed inputs are provided to the ELM.

Naturally, computation-accuracy of analog hardware systems with their inherent noise do not approach the accuracy reached with numerical simulations with arbitrary-precision variables. In the presence of noisy outputs the ELMs has a better performance than the ESNs. Experimental data are presented in Fig. 4 by star-symbols. Data was recorded using the experimental parameters introduced above. One has to keep in mind that for such hardware implementations a modification of the node number can not easily be implemented. Results are therefore restricted to *D* up to 800.

### Channel equalization

As a third task, we evaluate the performance of the system for the equalization of a wireless communication channel. This task has been previously used in the ESN community^4^,^8^. The input to the channel is an independent and identically distributed random sequence *s*(*i*) with values drawn from {−3, −1, 1, 3}. The channel is modeled as a linear system with memory followed by a memoryless noisy nonlinearity. Thus, the input *s*(*i*) first goes through the linear channel, yielding 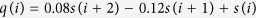

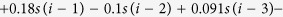

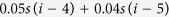

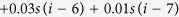
. It then goes through a noisy nonlinear channel, yielding 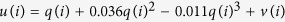, where *v*(*i*) is a Gaussian noise with zero mean adjusted in power to yield signal to noise ratios ranging from 16 to 28 dB. The task consists in reconstructing the input signal *s*(*i*) given the output sequence of the channel *u*(*i*). The performance on this task is measured using the Symbol Error Rate (SER), that is the fraction of inputs *s*(*n*) that are misclassified.

In the numerical simulations and experimental realizations, we take identical parameters, *κ* = 0.9, 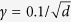, *ϕ* = 0.4*π*, *D* = 246 and *β* = 0 (ELM) or *β* = 1 (ESNs). We use 2000 samples for training, 6000 samples for validation and 6000 samples for testing.

**Figure 5:**
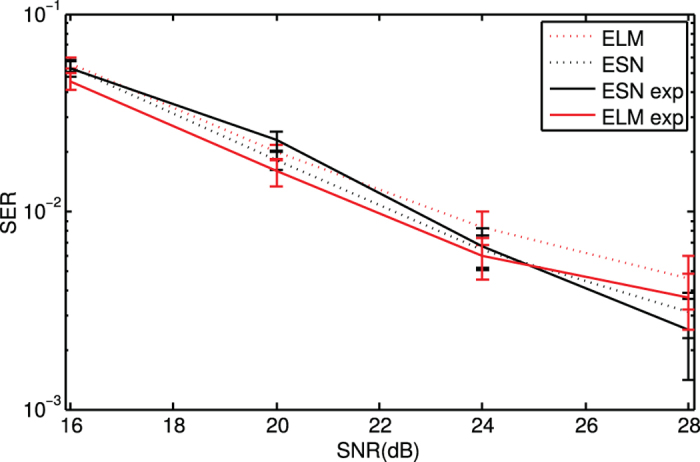
Symbol error rate (SER) for the channel equalization task as a function of the signal to noise ratio (SNR) in the channel. Results obtained with ESN (*d* = 1) and ELM (*d* = 7) for an experimental realization with 40 dB (solid lines) and the corresponding numerical simulations with noise (dotted lines). Discontinuous and continuous lines are mean values and error bars represent two times the standard deviation over 100 realizations.

Figure 5 illustrates the results for the ESN and ELM with *d* = 1 and *d* = 7, respectively. We compute the SER for the experimental condition with a signal to noise ratio of 40 dB and the corresponding numerical simulations with the same amount of noise. Overall, there is a good quantitative agreement between experimental and numerical results. As shown in Fig. 5, the SER rapidly decreases for an increasing channel signal to noise ratio. Although the numerical results of ESN are slightly better than those of ELM , in the experimental results this only happens for the case of lower channel SNR (28 dB). We numerically find that the best performance (lower SER) for ELMs are obtained with *d* ≥ 7. ELMs with *d* < 6 do not have sufficient memory to properly solve the equalization task.

### Cancer classification with microarrray data

Finally, we consider cancer classification using microarray data^21^. The microarray data contains usually thousands of attributes (genes), available only for a comparatively small number of patients (less than a hundred). Thus, the input-dimensionality is typically huge as compared with the number of instances available for training/testing. In fact, the high dimensionality of microarray data makes processing them a difficult task for many classification techniques (e.g. standard neural networks), which apply feature selection algorithms to reduce the input dimensionality.

Since there is no temporal dependence in this example, the microarray classification task does not require memory of previous inputs, therefore ESNs cannot benefit from their fading-memory feature. In contrast, ELMs are good candidates for these problems since the simplicity of the ELMs approach allows to process all the inputs without the selection features of other techniques.

In particular we consider two similar cases: Leukemia and diffuse large B-cell lymphoma (DLBCL), with 72 and 77 cases, respectively, and each one with 7129 genes. For convenience, the microarray dataset are normalized between 0 and 1 and divided by the square root of the input-dimensionality before our test, i.e., the gene expression of each patient has a maximum value of 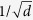 and a minimum value of 0.

**Figure 6:**
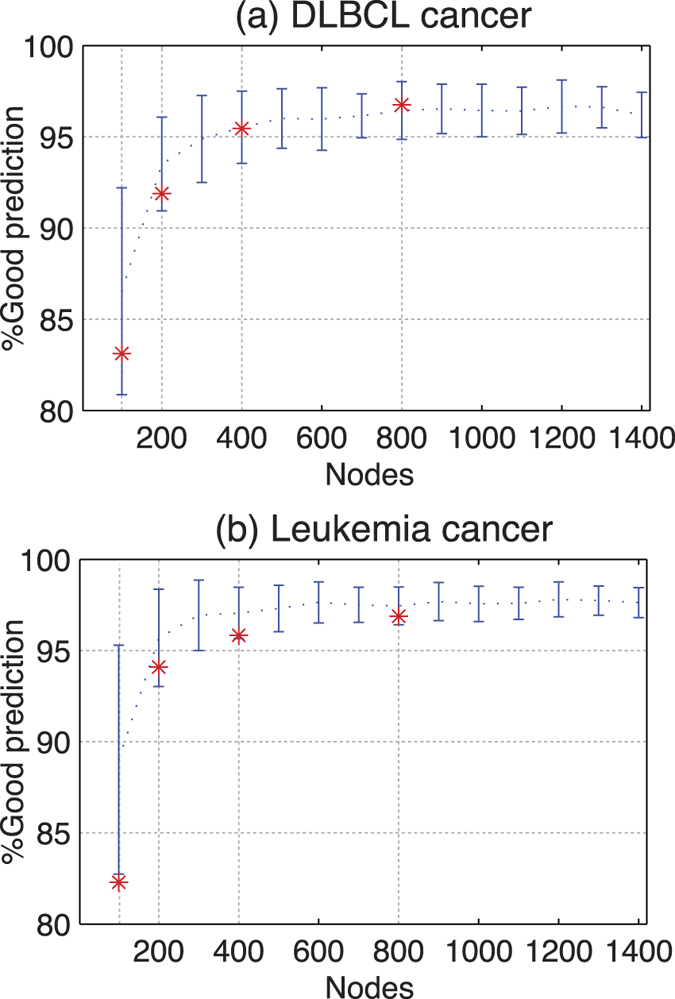
Percentage Success Rate in classification with ELMS vs. number of neurons (from 100 to 1400). Discontinuous blue line are mean values and error bars represent two times the standard deviation over 100 simulation results with difference random input projections. The red stars points are the experimental results averaging over 4 different random projections.

Figure 6 shows the results obtained from a Leave-One-Out (LOO) cross-validation. In both cases, we chose the parameters *γ* = 0.01 and *β* = 0. The average success rate in classification is higher than 95% if the number of virtual neurons is above 400. Similar results have been obtained using ELMs^22^,^23^. The experimental and the numerical results show a very good agreement. In this classification task, the noise-induced performance degradation seems to be negligible. This result is in agreement with previous findings, showing that classification tasks are less prone to noise degradation.

## Conclusions

In this work we have presented a unified framework for ELMs and ESNs based on a single nonlinear neuron with a feedback loop. In fact, activating or deactivating a single connection (the feedback connection of the neuron) we can easily switch between both hardware-implemented learning machines. In addition, the unified framework facilitates a better understanding of the fundamental similarities between both concepts.

A key point of this unified framework is that it enables a hardware implementation of ELMs and ESNs with an almost effortless mechanism that switches between both schemes. Consequently, we presented a hardware optoelectronic implementation that can be alternatively used as ELMs or ESNs. Moreover, this concept can be easily transferred to other hardware implementations based on a single time-delay neuron in electronic^7^, or all-optical systems^10^,^11^,^24^. None of the previously proposed hardware implementations of ELMs or ESNs based on multiple nonlinear neurons could implement both approaches without a complicated architecture.

The proposed photonic system retains the benefits provided by ESN implementations based on cyclic connection neurons structures, and at the same time has a simpler control structure to change and works as an ELM. Even though analog hardware-implementations are only emerging, they already demonstrate competitive performance. Especially all-optical implementations can lead the way to future, ultra-fast hardware systems of machine learning concepts. Nonlinear optical processes easily exhibit bandwidths of 100s of GHz and above. Furthermore, implementations based on inherent hardware-nonlinearities could allow for unachieved energy efficiency.

## Additional Information

**How to cite this article**: Ortín, S. *et al*. A Unified Framework for Reservoir Computing and Extreme Learning Machines based on a Single Time-delayed Neuron. *Sci. Rep*. **5**, 14945; doi: 10.1038/srep14945 (2015).
